# Baseline Predictors of Adverse Outcome and Survival in Adults Hospitalized with Invasive Pneumococcal Disease

**DOI:** 10.3390/healthcare14142057

**Published:** 2026-07-09

**Authors:** Sergio Venturini, Ingrid Reffo, Mariateresa Casarotto, Maria Teresa Bortolin, Giancarlo Basaglia, Francesco Cugini, Giovanni Del Fabro, Agnese Zanus-Fortes, Camilla Negri, Astrid Callegari, Federico Giovagnorio, Lucia Corich, Stefano Tavano, Umberto Zuccon

**Affiliations:** 1 Department of Infectious Diseases, ASFO Santa Maria degli Angeli Hospital of Pordenone, 33170 Pordenone, Italy; sergio.venturini@asfo.sanita.fvg.it (S.V.); giovanni.delfabro@asfo.sanita.fvg.it (G.D.F.); agnese.zanusfortes@asfo.sanita.fvg.it (A.Z.-F.); camilla.negri@asfo.sanita.fvg.it (C.N.); astrid.callegari@asfo.sanita.fvg.it (A.C.); federico.giovagnorio@asfo.sanita.fvg.it (F.G.); 2 Department of Anaesthesiology, ASFO Santa Maria dei Battuti Hospital of San Vito al Tagliamento, via Savorgnano, 2, San Vito al Tagliamento, 33078 Pordenone, Italy; 3 Department of Microbiology, ASFO Santa Maria degli Angeli Hospital of Pordenone, 33170 Pordenone, Italy; mariateresa.casarotto@asfo.sanita.fvg.it (M.C.); mariateresa.bortolin@asfo.sanita.fvg.it (M.T.B.); giancarlo.basaglia@asfo.sanita.fvg.it (G.B.); lucia.corich@asfo.sanita.fvg.it (L.C.); 4 Department of Emergency Medicine, ASUFC Hospital of San Daniele, 33038 San Daniele del Friuli, Udine, Italy; francesco.cugini@asufc.sanita.fvg.it; 5 Department of Pulmonology, ASFO Santa Maria degli Angeli Hospital of Pordenone, 33170 Pordenone, Italy; stefano.tavano@asfo.sanita.fvg.it (S.T.); umberto.zuccon@asfo.sanita.fvg.it (U.Z.)

**Keywords:** *Streptococcus pneumoniae*, invasive pneumococcal disease, prognostic markers, procalcitonin, pH, bacteremia

## Abstract

**Highlights:**

**What are the main findings?**

- In hospitalized adults with invasive pneumococcal disease, non-survivors showed lower baseline pH, higher procalcitonin concentrations, and more frequent invasive mechanical ventilation than survivors.
- In time-to-event analysis, lower pH showed the most consistent prognostic signal for 65-day mortality after adjustment for age, sex, procalcitonin, and invasive mechanical ventilation.

**What are the implications of the main findings?**

- Early acid-base derangement may provide a pragmatic bedside marker to support risk stratification in adults hospitalized with invasive pneumococcal disease.
- Procalcitonin and the need for ventilation should be interpreted as complementary markers of acute disease severity rather than definitive independent predictors.

**Abstract:**

Background/Objectives: Invasive pneumococcal disease (IPD) remains associated with substantial mortality. We evaluated whether routinely available baseline variables were associated with 65-day all-cause mortality among adults hospitalized with IPD. Methods: We conducted a retrospective cohort study of 195 adults hospitalized with IPD. The primary endpoint was 65-day all-cause mortality, with hospital admission as the time origin and administrative censoring at day 65. Logistic regression and Cox proportional hazards models were fitted with a prespecified adjustment set that included age, sex, invasive mechanical ventilation, procalcitonin, and pH. Results: Overall, 42 patients died during follow-up. Non-survivors had lower admission pH, higher procalcitonin concentrations, and more frequent invasive mechanical ventilation. In the adjusted logistic model, lower pH showed a directionally consistent but non-significant association with mortality (OR per 0.05-unit decrease, 1.45; 95% CI, 0.96–2.20; *p* = 0.080). In the adjusted Cox model, lower pH was associated with a higher mortality hazard (HR per 0.05-unit decrease, 1.31; 95% CI, 1.03–1.65; *p* = 0.026). When lower pH was correctly oriented as the risk marker, its AUC was 0.729. The apparent AUC of the multivariable model was 0.831, and the bootstrap optimism-corrected AUC was 0.785. Conclusions: In adults hospitalized with IPD, lower admission pH, higher procalcitonin levels, and invasive mechanical ventilation were associated with worse outcomes. Lower pH showed the most consistent prognostic signal, particularly in time-to-event analysis, but estimates were imprecise and should be interpreted as exploratory. These findings support the potential bedside value of early acid–base assessment for risk stratification in IPD.

## 1. Introduction

Invasive pneumococcal disease (IPD) remains a leading cause of severe infection and mortality worldwide, despite advances in vaccination, antimicrobial therapy, and critical care support [1,2]. IPD is defined by the isolation of Streptococcus pneumoniae from normally sterile sites, most commonly blood or cerebrospinal fluid (CSF). Its clinical presentation ranges from bacteremic pneumonia to meningitis and primary bloodstream infection [3]. Although the incidence of IPD has decreased in many countries following the introduction of pneumococcal conjugate vaccines, the disease continues to impose a significant clinical burden, particularly among older adults and those with chronic comorbidities or impaired immune function [3].

Mortality rates associated with IPD remain substantial. Systematic reviews have reported case-fatality rates approaching 20% among hospitalized adults, with higher mortality observed in cases presenting with septic shock, respiratory failure, or central nervous system involvement [1]. Prognosis depends on a complex interplay of host factors, acute physiological derangements, microbial characteristics, and access to timely supportive care [4]. Several studies have identified advanced age, immunosuppression, alcohol misuse, chronic cardiopulmonary disease, and septic shock as adverse prognostic indicators [4,5,6,7]. More recently, attention has also been directed toward the influence of pneumococcal serotype, vaccination status, antimicrobial susceptibility, and severity-of-illness scores [8,9].

While composite prognostic tools can aid in risk assessment, their clinical utility may be limited by complexity, delayed results, or the need for multiple variables not immediately available at the bedside [10,11,12]. Consequently, there is growing interest in identifying simple, readily accessible markers to support early risk stratification during initial patient evaluation [5,6,13].

Among potential markers, acid–base status warrants particular attention. Admission pH reflects the integrated physiological impact of respiratory dysfunction, tissue hypoperfusion, metabolic disturbances, and compensatory responses. Even modest deviations within the normal reference range may provide valuable insight into physiological reserve and disease severity [5]. Similarly, procalcitonin (PCT) and the need for invasive mechanical ventilation (IMV) may reflect distinct but complementary dimensions of acute illness severity and the requirement for organ support [13].

However, the prognostic significance of these readily available bedside variables has not been extensively studied specifically in cohorts of adults hospitalized with IPD. Most previous studies have focused on demographic factors, comorbidities, microbiological features, or composite severity scores, rather than on routine physiological and laboratory parameters measured at presentation [4,5,6].

Therefore, this study aimed to evaluate baseline predictors of 65-day all-cause mortality in adults hospitalized with IPD. We also explored the association between routinely available clinical and laboratory variables and survival over time. We hypothesized that early physiological disturbances—such as lower admission pH, higher procalcitonin levels, and the need for invasive mechanical ventilation—would be associated with worse survival outcomes.

## 2. Materials and Methods

### 2.1. Study Design and Setting

This retrospective observational cohort study was conducted across three hospitals within the Azienda Sanitaria Friuli Occidentale (ASFO) healthcare network (Pordenone, San Vito al Tagliamento, and Spilimbergo, Italy). Consecutive adult patients diagnosed with invasive pneumococcal disease (IPD) between January 2018 and December 2024 were identified from institutional microbiological databases and electronic medical records.

### 2.2. Study Population

IPD was defined as the isolation of *Streptococcus pneumoniae* from a normally sterile body site, including blood, CSF, pleural fluid, or other sterile-site specimens. All available isolates were submitted to the Italian National Institute of Health (Istituto Superiore di Sanità) for confirmatory serotyping when feasible.

Adult patients (≥18 years) hospitalized with microbiologically confirmed IPD were eligible for inclusion. When multiple positive sterile-site samples were obtained during the same clinical episode, the episode was counted once, while all positive sampling sites were recorded.

### 2.3. Data Collection

Demographic, clinical, respiratory, laboratory, microbiological, and outcome variables were extracted from routinely collected electronic medical records.

Baseline variables included age, sex, vaccination status, smoking history, requirement for invasive mechanical ventilation (IMV) at initial presentation, oxygenation indices, arterial blood gas measurements, inflammatory biomarkers, hematological parameters, renal function indices, and serum biochemical measurements. Laboratory and physiological variables were defined as the first available values obtained during the initial clinical assessment after hospital admission.

Pneumococcal serotypes and vaccine coverage status were recorded when available. Because serotyping was not available for all patients, serotype analyses were considered exploratory.

### 2.4. Outcome

The primary outcome was 65-day all-cause mortality.

Hospital admission served as the time origin for all analyses. Follow-up was administratively censored at 65 days. Patients alive at the end of follow-up were treated as censored observations, whereas patients who died during follow-up were assigned the observed time from admission to death.

### 2.5. Statistical Analysis

Continuous variables are presented as median and interquartile range (IQR), whereas categorical variables are reported as counts and percentages. Comparisons between survivors and non-survivors were performed using the Wilcoxon rank-sum test for continuous variables and Fisher’s exact test for categorical variables.

Associations between baseline variables and mortality were initially explored using univariable logistic regression models. A prespecified multivariable logistic regression model was subsequently fitted to include age, sex, IMV, admission procalcitonin (PCT), and admission pH. Because of the limited number of outcome events, Firth penalized logistic regression was performed as a sensitivity analysis to reduce small-sample bias.

To improve clinical interpretability, pH was rescaled and modeled as a 0.05-unit decrease.

Discriminative performance was assessed using receiver operating characteristic (ROC) curve analysis. For pH, lower values were considered the risk direction. Apparent model discrimination was quantified using the area under the ROC curve (AUC), and internal validation was explored using bootstrap optimism correction.

Time-to-event analyses were conducted using Kaplan–Meier estimates and Cox proportional-hazards regression models. The same prespecified adjustment set was applied to multivariable Cox analyses. Results are reported as hazard ratios (HRs) with 95% confidence intervals (CIs). The proportional-hazards assumption was evaluated using Schoenfeld residuals.

Missing data were handled using complete-case analysis for multivariable models. As an exploratory secondary analysis, serotype distributions were summarized both overall and by survival status. The most frequent serotypes were retained as separate categories, whereas less frequent serotypes were grouped as “Other.” Given the proportion of missing serotype data and the limited number of events in each category, serotype analyses were considered descriptive and hypothesis-generating.

All analyses were conducted in R version 4.5.2 (R Foundation for Statistical Computing, Vienna, Austria). Two-sided *p*-values < 0.05 were considered statistically significant.

### 2.6. Ethical Considerations

Ethical review and formal approval were not required because of the retrospective observational design and the use of pseudonymized routinely collected clinical data. Data processing was conducted in accordance with applicable European and Italian data protection regulations, including Regulation (EU) 2016/679 and Legislative Decree No. 196/2003, as amended. At hospital admission, patients or their legal representatives provided standard institutional consent for the processing of health data in accordance with institutional procedures.

## 3. Results

Among the 195 IPD cases, *S. pneumoniae* was isolated from blood only in 172 patients (88.2%), from CSF only in 9 (4.6%), and from both blood and CSF in 14 (7.2%). During the 65-day follow-up, 42 patients (21.5%) died, and 153 (78.5%) were alive at administrative censoring. Baseline characteristics by 65-day survival status are shown in Table 1. The median age was 70.84 years (IQR 54.61–81.36). Compared with survivors, non-survivors had lower admission pH, higher procalcitonin (PCT) concentrations, lower body temperature, and more frequent invasive mechanical ventilation (IMV). Median pH was 7.47 (IQR 7.41–7.49) in survivors and 7.41 (IQR 7.36–7.46) in non-survivors (*p* < 0.001). The median PCT was 4.31 µg/L (IQR 0.71–17.82) in survivors and 23.42 µg/L (IQR 5.33–47.30) in non-survivors (*p* = 0.002). IMV was used in 9.8% of survivors and 23.8% of non-survivors (*p* = 0.033).

In univariable logistic regression, higher PCT, lower pH, higher creatinine, lower body temperature, and IMV were associated with 65-day mortality. To improve interpretability, pH was modeled per 0.05-unit decrease. In the prespecified multivariable logistic model fitted in the complete-case analysis, lower pH showed a directionally consistent but non-significant association with 65-day mortality (OR per 0.05-unit decrease: 1.45; 95% CI, 0.96–2.20; *p* = 0.080). IMV was associated with higher odds of mortality (OR 3.55; 95% CI, 0.97–13.28; *p* = 0.054), whereas PCT showed a weaker positive association (OR per 1 µg/L increase, 1.02; 95% CI, 1.00–1.04; *p* = 0.062). Firth penalized logistic regression yielded directionally similar results; the association between pH and mortality remained non-significant (*p* = 0.085) (Table 2).

**Figure 1:**
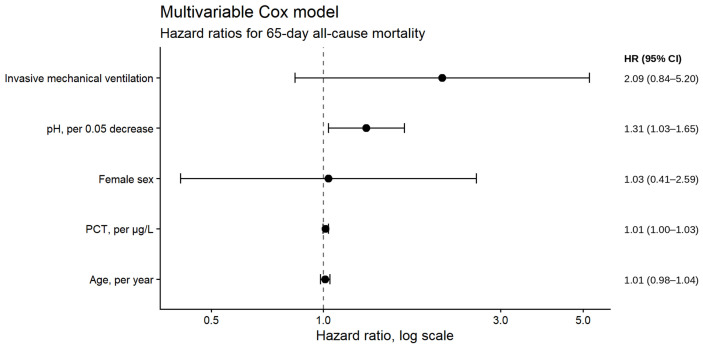
Adjusted hazard ratios for 65-day all-cause mortality from the prespecified multivariable Cox proportional hazards model. Hazard ratios (HRs) and 95% confidence intervals from the prespecified multivariable Cox model including age, sex, invasive mechanical ventilation, procalcitonin (PCT), and admission pH. The dashed vertical line indicates the null-effect line, corresponding to an odds ratio of 1. Horizontal lines represent 95% confidence intervals. Age was modeled per 1-year increase, PCT per 1 µg/L increase, and pH per 0.05-unit decrease. The vertical dashed line indicates the null value (HR = 1.0). Lower admission pH showed the most consistent association with mortality hazard during follow-up.

In time-to-event analyses, lower pH, higher PCT, and IMV were associated with worse survival in univariable Cox models. In the prespecified multivariable Cox model, lower pH remained associated with higher mortality hazard (HR per 0.05-unit decrease, 1.31; 95% CI, 1.03–1.65; *p* = 0.026). PCT (HR 1.01; 95% CI, 1.00–1.03; *p* = 0.079) and IMV (HR 2.09; 95% CI, 0.84–5.20; *p* = 0.113) were directionally consistent but attenuated after adjustment. Age and sex were not independently associated with mortality hazard (Table 2 and Figure 1). The proportional-hazards assumption was not violated.

**Figure 2:**
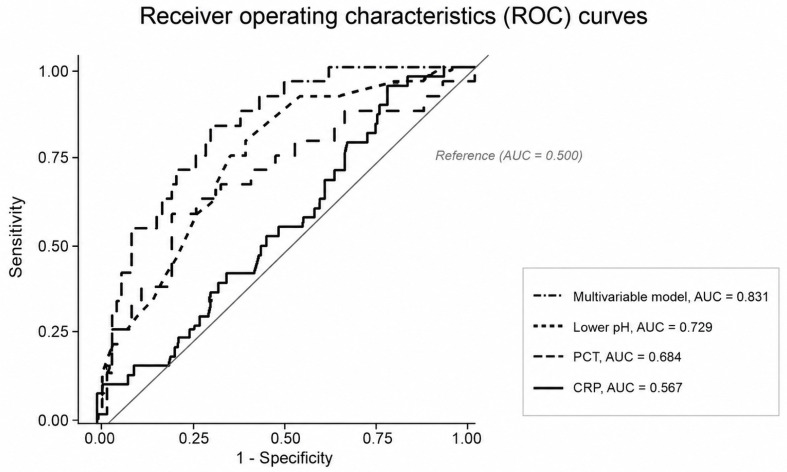
Receiver operating characteristic (ROC) curves for 65-day all-cause mortality. The plot displays the discriminatory performance of the prespecified multivariable model, admission pH, procalcitonin (PCT), and C-reactive protein (CRP). For pH, lower values were oriented to indicate higher mortality risk. The diagonal line represents discrimination no better than chance (AUC = 0.500).

The multivariable model showed good apparent discrimination for 65-day mortality, with an AUC of 0.831. After bootstrap optimism correction, the AUC was 0.785. When lower pH was correctly oriented as the risk marker, the AUC was 0.729. The AUC for PCT was 0.684, and that for C-reactive protein was 0.567 (Figure 2).

**Figure 3:**
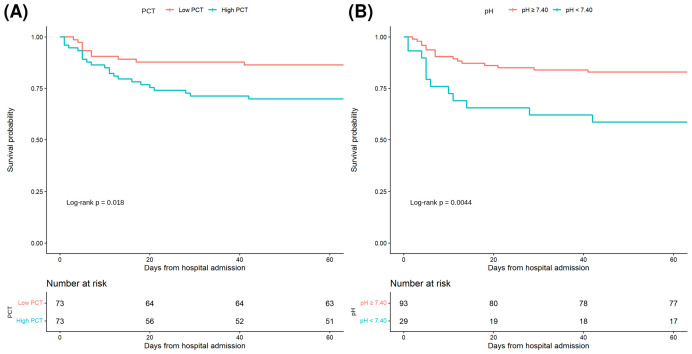
Kaplan–Meier survival curves according to admission procalcitonin and pH. (**A**) Kaplan–Meier estimates of 65-day survival, stratified by admission procalcitonin (PCT) concentration above or below the cohort median. Patients with higher PCT concentrations had significantly lower survival during follow-up (log-rank *p* = 0.018). (**B**) Kaplan–Meier estimates of 65-day survival, stratified by admission pH. Patients with pH < 7.40 had significantly lower survival than those with pH ≥ 7.40 (log-rank *p* = 0.004). Numbers at risk are shown below each panel.

In unadjusted Kaplan–Meier analyses, patients with higher admission PCT concentrations and those with admission pH < 7.40 had lower 65-day survival (Figure 3).

**Figure 4:**
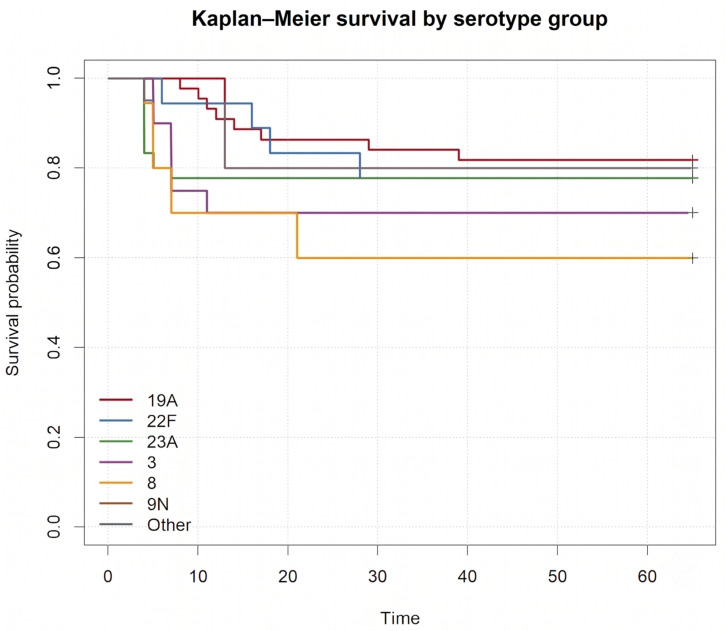
Kaplan–Meier estimates of 65-day survival by pneumococcal serotype group. The analysis included 112 of 195 patients (57.4%) with available serotype data. Kaplan–Meier curves are shown for the most frequent serotype groups retained as separate categories (19A, 22F, 23A, 3, 8, and 9N), with all other observed serotypes grouped as “Other.” The 9N curve is present but partly overlaps with the “Other” curve during follow-up, which may reduce its visual distinction in the plot. Because of the limited number of patients and deaths within individual serotype groups, as well as the proportion of missing serotype data, this analysis should be considered exploratory.

Vaccination and serotype analyses were exploratory due to missing data and limited sample sizes within individual categories. Serotype data were available for 112 of 195 patients (57.4%). The most frequent serotypes were 3, 8, 22F, and 23A. No serotype group showed a clear association with 65-day mortality. Given the limited number of events within individual serotype categories and the proportion of missing serotype data, these analyses should be interpreted as descriptive and hypothesis-generating only (Figure 4).

## 4. Discussion

In this retrospective cohort of adults hospitalized with IPD, the findings extend the previous prognostic literature by highlighting the complementary information provided by routinely available measures of early physiological derangement. Previous studies have mainly emphasized host characteristics, comorbidities, microbiological factors, and composite severity indicators [1,2,7,8]. The contribution of the present study is therefore not the identification of admission pH as a stand-alone prognostic biomarker, but the IPD-specific observation that acid–base status may serve as an integrated measure of acute physiological disturbance alongside inflammatory and organ-support variables. Lower admission pH showed the most consistent prognostic signal, particularly in time-to-event analysis, whereas PCT and IMV appeared to reflect complementary and partially overlapping dimensions of disease severity. Given the observational design and limited sample size, these findings should be considered exploratory.

The association between lower pH and mortality is biologically and clinically plausible. Arterial pH integrates the effects of respiratory dysfunction, tissue hypoperfusion, metabolic disturbances, renal compensation, and physiological reserve. Therefore, even modest reductions within or near the conventional reference range may identify patients with reduced capacity to compensate for severe infection [5,14]. In this context, pH should be interpreted as a nonspecific indicator of disrupted physiological homeostasis rather than as an IPD-specific or causal biomarker. The consistent direction of the association across analyses supports further investigation; however, the estimate should be interpreted cautiously, given the narrow biological range of pH, missing data, and the limited number of complete cases.

PCT and IMV may provide complementary, although less specific, prognostic information. Higher PCT concentrations may reflect greater systemic inflammatory activation and more severe invasive bacterial infection [11,15,16,17,18], whereas the need for IMV identifies patients with advanced respiratory failure, impaired gas exchange, or global clinical instability [2,13]. The attenuation of both associations after adjustment suggests that their prognostic information overlaps with other components of acute disease severity. Neither variable should therefore be interpreted as an independent causal determinant of mortality; rather, both may contribute to a multidimensional assessment that incorporates inflammatory burden, physiological disturbance, organ-support requirements, and clinical trajectory.

The exploratory serotype analysis did not show a clear association with 65-day mortality. Serotype data were unavailable for a substantial proportion of patients, and the number of deaths within individual serotype groups was small. Therefore, these findings should be considered descriptive and hypothesis-generating. More comprehensive microbiological and vaccination data will be required to determine whether specific serotypes independently contribute to prognosis in adult IPD [7,19].

Our results indicate that routinely collected admission variables can provide useful insights for early risk assessment in adults hospitalized with IPD. Nonetheless, these variables should not replace comprehensive clinical evaluations, comorbidity assessments, or validated severity scoring systems. Clinically, admission pH should not be used as a standalone prognostic indicator or as an immediate basis for treatment or decision-making, but it should complement overall judgment. The added prognostic value of admission pH beyond existing tools still needs validation in adequately powered studies.

This study has several limitations. First, its retrospective design precludes causal inference and leaves the analysis vulnerable to residual confounding. Second, several clinically relevant variables, including comorbidity burden, frailty, immunosuppression, shock, vasopressor use, lactate, treatment limitations, and validated severity scores, were not systematically available. Third, missing data were substantial for several laboratory and microbiological variables, so multivariable analyses were based on complete cases. This reduced the effective sample size and may have introduced selection bias. Fourth, the number of deaths was modest relative to the number of predictors, resulting in imprecise estimates and a risk of overfitting despite the use of a prespecified adjustment set and a Firth-penalized sensitivity analysis. Fifth, the apparent AUC of the multivariable model was estimated on the derivation dataset; although a bootstrap optimism correction was applied, external validation was not available. Sixth, measurement timing relied on routinely collected clinical data and may not have been fully standardized. Finally, the study period spanned 2018 to 2024, during which vaccination coverage, circulating serotypes, diagnostic practices, antimicrobial and supportive management, and healthcare organization may have changed. The limited number of outcome events precluded reliable adjustment for calendar period or stratification by vaccination era; therefore, residual temporal confounding cannot be excluded.

## 5. Conclusions

In adults hospitalized with IPD, lower admission pH showed the most consistent association with 65-day mortality, whereas higher PCT concentrations and the need for IMV provided complementary indicators of acute disease severity. These exploratory findings suggest that early acid–base assessment may contribute to multidimensional prognostic evaluation, but do not support the use of pH as a stand-alone marker or as a treatment threshold. Larger prospective studies incorporating temporal, microbiological, vaccination-related, and clinical management factors are needed to validate these observations and determine their incremental prognostic value.

## Figures and Tables

**Table 1:** Baseline characteristics according to 65-day survival status.

Variable	Overall (*n* = 195)	Survivors (*n* = 153)	Non-Survivors (*n* = 42)	*p* Value
Demographic, clinical, and microbiological characteristics				
Age, years	70.84 [54.61–81.36]	70.19 [54.59–80.02]	76.50 [60.01–85.87]	0.142
Female sex	108 (55.4%)	81 (52.9%)	27 (64.3%)	0.222
Smoking status				0.357
Non-smoker	37 (19.0%)	29 (19.0%)	8 (19.0%)	
Former smoker	26 (13.3%)	19 (12.4%)	7 (16.7%)	
Current smoker	33 (16.9%)	23 (15.0%)	10 (23.8%)	
Unknown	99 (50.8%)	82 (53.6%)	17 (40.5%)	
Pneumococcal vaccination	93 (47.7%)	73 (47.7%)	20 (47.6%)	1.000
Vaccine-covered serotype *	20 (40.8%)	14 (36.8%)	6 (54.5%)	0.320
Invasive mechanical ventilation	25 (12.8%)	15 (9.8%)	10 (23.8%)	0.033
Adequate antibiotic therapy †	186 (96.4%)	147 (97.4%)	39 (92.9%)	0.176
Corticosteroid therapy †	86 (44.6%)	66 (43.7%)	20 (47.6%)	0.726
Physiological and respiratory variables				
Body temperature, °C	38.00 [37.27–39.00]	38.10 [37.45–39.00]	37.70 [36.80–38.30]	0.006
PaO_2_/FiO_2_, mmHg	242.50 [170.75–306.25]	254.00 [173.50–305.50]	233.00 [146.00–314.00]	0.732
SaO_2_, %	93.35 [91.05–95.97]	93.35 [91.15–95.82]	93.30 [91.15–96.75]	0.786
Arterial pH	7.46 [7.40–7.49]	7.47 [7.41–7.49]	7.41 [7.36–7.46]	<0.001
PaCO_2_, mmHg	31.75 [28.80–35.70]	32.45 [28.80–35.70]	30.70 [26.65–35.72]	0.512
PaO_2_, mmHg	62.75 [55.65–69.77]	61.30 [55.00–67.80]	66.30 [58.50–72.00]	0.135
Laboratory variables				
C-reactive protein, mg/dL	18.05 [7.92–29.38]	17.60 [6.80–29.30]	21.50 [11.55–29.90]	0.198
Procalcitonin, µg/L	6.51 [1.03–26.77]	4.31 [0.71–17.82]	23.42 [5.33–47.30]	0.002
White blood cell count, 10^3^/µL	13.70 [8.94–18.96]	14.06 [9.02–18.59]	12.86 [8.43–20.80]	0.933
Creatinine, mg/dL	1.09 [0.82–1.52]	1.08 [0.83–1.50]	1.13 [0.78–2.40]	0.313
Platelet count, ×10^9^/L	197.00 [138.25–253.75]	197.00 [126.00–257.00]	202.00 [155.00–236.00]	0.884
Glucose, mg/dL	136.00 [102.00–182.25]	136.00 [102.00–193.00]	136.00 [106.00–149.00]	0.254

Values are presented as median [interquartile range] or *n* (%). Percentages were calculated using available data. Survival status refers to vital status at day 65. *p* values compare survivors and non-survivors using the Wilcoxon rank-sum test for continuous variables and Fisher’s exact test for categorical variables, as appropriate. The *p* value for smoking status represents an overall comparison across all four categories. * Vaccine-covered serotype was assessed in 49 patients with available serotype and vaccine-coverage data (38 survivors and 11 non-survivors). † Treatment data were available for 193 patients (151 survivors and 42 non-survivors). PaO_2_, arterial oxygen tension; FiO_2_, fraction of inspired oxygen; SaO_2_, arterial oxygen saturation; PaCO_2_, arterial carbon dioxide tension.

**Table 2:** Prespecified multivariable regression models for 65-day all-cause mortality.

Variable	Logistic OR OR (95% CI); *p* Value	Firth Logistic OR OR (95% CI); *p* Value	Cox HR HR (95% CI); *p* Value
Age, per year	1.03 (0.99–1.07) *p* = 0.145	1.02 (0.99–1.06) *p* = 0.156	1.01 (0.98–1.04) *p* = 0.512
Female sex	1.62 (0.55–5.10) *p* = 0.392	1.54 (0.55–4.57) *p* = 0.414	1.03 (0.41–2.59) *p* = 0.950
Invasive mechanical ventilation	3.55 (0.97–13.28) *p* = 0.054	3.31 (0.97–11.58) *p* = 0.057	2.09 (0.84–5.20) *p* = 0.113
Procalcitonin, per µg/L	1.02 (1.00–1.04) *p* = 0.062	1.02 (1.00–1.04) *p* = 0.066	1.01 (1.00–1.03) *p* = 0.079
pH, per 0.05-unit decrease	1.45 (0.96–2.20) *p* = 0.080	1.33 (0.96–1.85); *p* = 0.085	1.31 (1.03–1.65) *p* = 0.026

Values are adjusted odds ratios (ORs) or hazard ratios (HRs) with 95% confidence intervals (CIs) and *p* values. All models included age, sex, invasive mechanical ventilation, admission procalcitonin, and admission pH. Age was modeled per 1-year increase, procalcitonin per 1 µg/L increase, and pH per 0.05-unit decrease. Firth penalized logistic regression was performed as a sensitivity analysis to reduce small-sample bias.

## Data Availability

The data presented in this study are available on request from the corresponding author.
